# Protein Engineering in the Design of Protein–Protein Interactions: SARS-CoV-2 Inhibitors as a Test Case

**DOI:** 10.1021/acs.biochem.1c00356

**Published:** 2021-07-01

**Authors:** Jiří Zahradník, Gideon Schreiber

**Affiliations:** Department of Biomolecular Sciences, Weizmann Institute of Science, Rehovot 76100, Israel

## Abstract

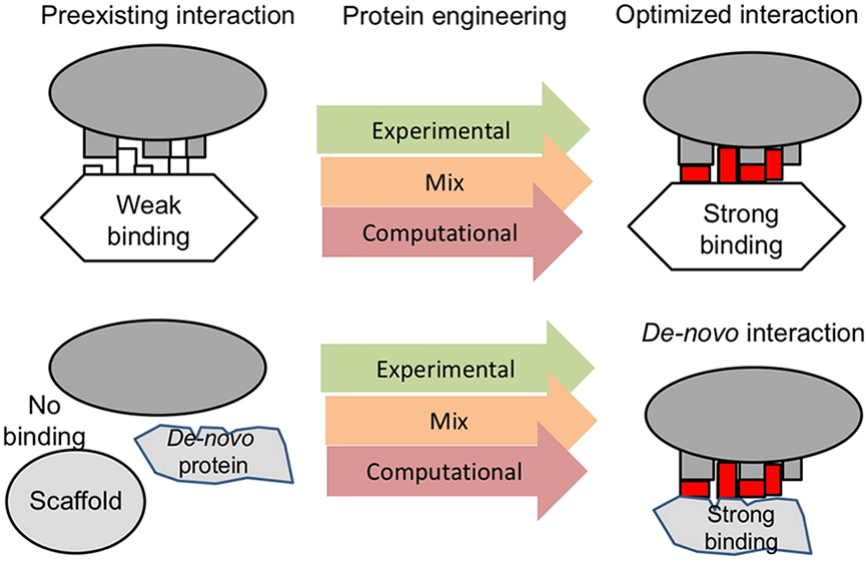

The formation of specific protein–protein interactions (PPIs) drive most biological processes. Malfunction of such interactions is the molecular driver of many diseases. Our ability to engineer existing PPIs or create new ones has become a vital research tool. In addition, engineered proteins with new or altered interactions are among the most critical drugs that have been developed in recent years. These include antibodies, cytokines, inhibitors, and others. Here, we provide a perspective on the current status of the methods used to engineer new or altered PPIs. The emergence of the COVID-19 pandemic, which resulted in a worldwide quest to develop specific PPI inhibitors as drugs, provided an up-to-date and state-of-the-art status report on the methodologies for engineering PPIs targeting the interaction of the viral spike protein with its cellular target, ACE2. Multiple, very high affinity binders were generated within a few months using *in vitro* evolution by itself, or in combination with computational design. The different experimental and computational methods used to block this interaction provide a road map for the future of PPI engineering.

The formation of specific interactions between proteins within the crowded milieu of the living organism is crucial for all aspects of life. Proteins interact with other proteins to form signaling networks, to drive the immune response, to control transcription and translation, to regulate enzyme activity, and much more. Due to their large, heterogeneous surfaces, protein interactions can bind quickly, tightly, and specificaly to their partners, even in environments with a large number of competing noncognate molecules. This happens at an incredible range of concentrations, from millimolar to femtomolar. With protein–protein interactions (PPIs) being paramount in all aspects of life, it is not surprising that their malfunction is a driver of many diseases. At the same time, they have become a major source for drug development. Proteins forming specific interactions to modify biological processes are now the hottest selling new drugs globally. Five of the ten top-selling drugs (by value) are biologicals (proteins, mainly antibodies), which act by forming specific protein interactions. This is a result of the massive progress in protein engineering that has been achieved during the past 40 years when protein engineering was at its humble start.

## From History to Current Perspectives

Protein engineering started with the redesign of proteins to understand enzyme mechanisms, protein structure, and folding.^1−3^ From the early days, it was envisioned that the ability to design protein molecules would open a path to the fabrication of devices of complex atomic specifications. Engineering existing PPIs for higher affinity or creating new interactions was between the first applications of protein engineering. Nature invented protein engineering hundreds of millions of years ago, with the development of small antibodies. Kohler and Milstein^4^ applied the technology of antibody engineering for the production of mouse monoclonal antibodies by hybridoma technology and by this opened the door to engineer binders by need. However, they let nature make the selection, as understanding protein structure–function relations was still in its infancy.

A fundamental requirement for designing new or enhanced protein–protein interactions is understanding the nature of protein–protein interfaces. Natural protein–protein interfaces show a complementarity of only 70–75% between the surfaces of the partners, with the rest being occupied by water molecules within the interface.^5−7^ The overall architecture of protein–protein binding sites was suggested to include a hydrophobic, water-shielded interface core, surrounded by polar residues that provide specificity. However, different proteins show very different modes of interaction. Thus, the use of rules here is much more limited than for protein folding, where one always finds a hydrophobic core and polar surface (which may be why the design of *de novo* proteins seems to be a more straightforward task).

One of the troubling aspects hampering our efforts to tailor PPIs to our needs is the lack of knowledge about the effects of individual mutations on binding. Most studies applied alanine scanning mutagenesis,^8^ which provide information about the deletion of a specific amino acid (toward alanine) but not about the effect of substituting one amino acid with another one. This approach was revolutionized by the rise of so-called deep mutational scanning.^9^ In an elegant example by Heyne,^10^ they combined protein randomization, yeast surface display, deep sequencing, and few experimentally measured *K*_D_ data points. This resulted in the generation of binding data for all possible mutations within the interface between two proteins, BPTI and bovine trypsin. This kind of data is a gold mine for tuning force fields for PPI design, a task that current algorithms fail to accurately predict.^11−13^

When engineering a protein interface, we should first ask ourselves how unique its composition is. Previously, we examined the plasticity of the interface of TEM1-β-lactamase with its protein inhibitor BLIP and showed that most interfacial residues could be mutated without a loss of binding affinity, protein stability, or enzymatic activity, suggesting plasticity in the interface composition supporting high-affinity binding.^14^ Moreover, using random mutagenesis has shown that most proteins can form high-affinity PPIs with many other partners by introducing a small number of mutations.^15−17^ These findings clearly show that PPIs are not unique. Moreover, as the gap between protein thermostability and its working conditions increases, it allows for introduction of more mutations with destabilizing properties yet generating new PPIs without negatively affecting its structural integrity.^16,18^ Thus, prestabilization of a protein makes it easier for engineering, including PPIs.

Single-mutation changes provide only a partial picture of the energetics within a protein–protein interface. An early, groundbreaking study by Wells^19^ has shown that the effect of mutations on binding is additive within PPIs. This study was further refined by a study showing that PPI interfaces are organized in a modular manner, with a module comprising several residues from both binding partners that form a continuous network of interactions. The additivity of mutation was found to hold for residues located in different modules, while within modules, there is significant cooperativity between residues.^20^ This led to a design principle in which complete interface modules were replaced, which resulted in the design of the specificity of binding for similar interfaces.^21^ Along the same lines, binding specificity was also achieved together with high affinity by extending the interface to include a new specificity module.^22^ Further development in PPI specificity design was demonstrated by Netzer et al.,^23^ who aimed to design new high-specificity colE-immunoprotein pairs on top of the known interaction between these two proteins. Using a multistep design, they achieved pairwise specificity switches of >3 orders of magnitude relative to at least one of the noncognate proteins. They suggest that preorganized backbone conformations were more likely to result in high-specificity binding, providing a guideline for specificity design.

## Experimental Tools for Engineering Protein–Protein Interactions

**Figure 1:**
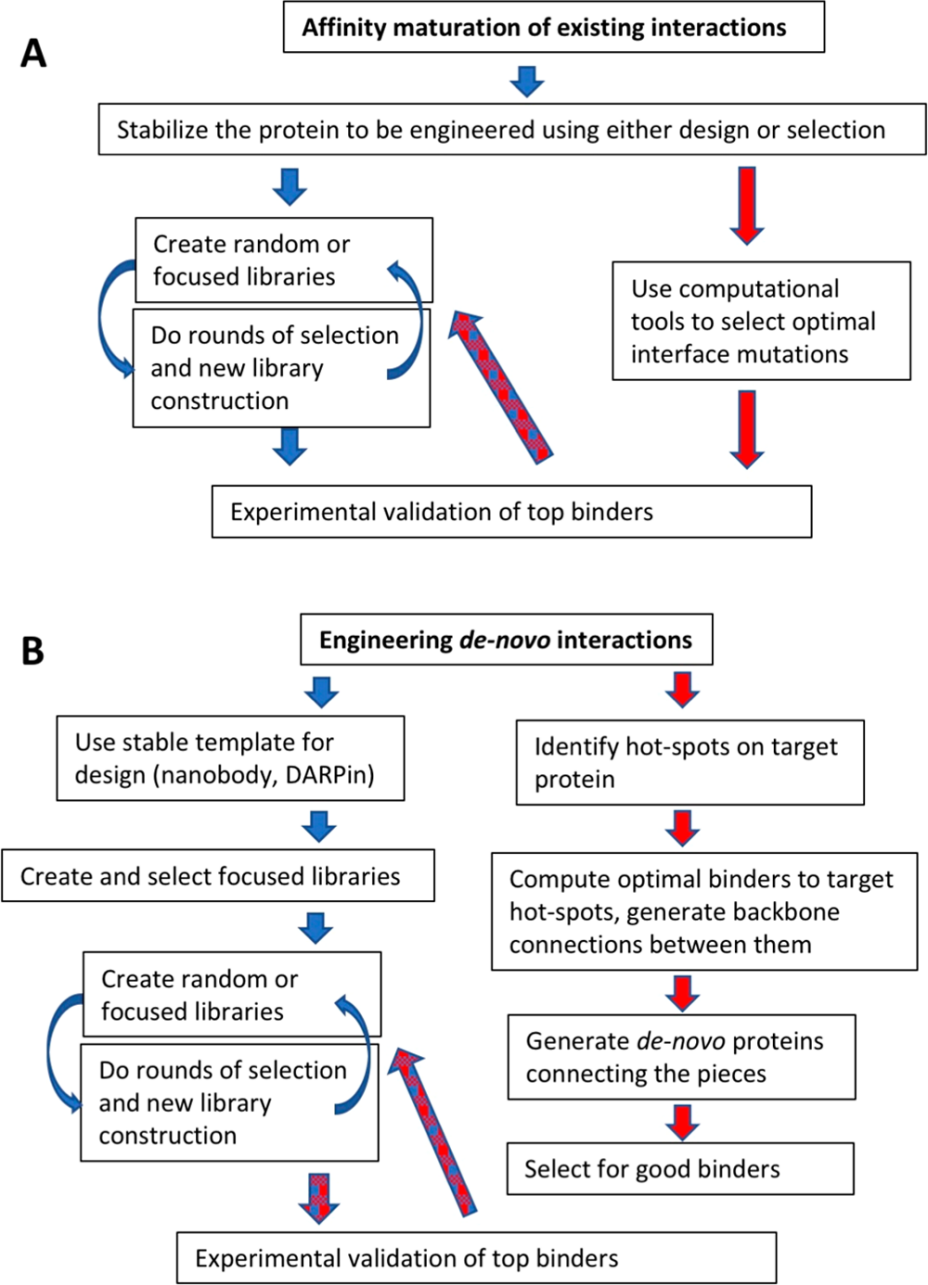
Flow diagrams for engineering altered or new PPIs. (A) To improve the binding affinity of an existing interaction, it is preferential to stabilize the prey protein to allow for a larger mutation space to be accommodated. This is followed by multiple rounds of *in vitro* selection or computing more favorable interactions. In most cases, the latter will also include a final step of selection of a designed focused library to achieve very high affinity. (B) To generate a binding protein from scratch, it is most common to use existing stable templates, which will undergo multiple rounds of in vitro evolution. For computational design of a new binding protein, the hot spots on the target protein are first identified. This is followed by computing backbone connections, which is the basis for designing mini-proteins. These are selected for binding and then undergo *in vitro* evolution to obtain the best binders.

Alanine scanning mutagenesis proved to be a very useful tool in pointing toward the most critical residues within a protein interface. These are now called “hot spots”, which refer to residues that upon mutation decrease the level of binding by >10-fold.^24^ Thus, knowing the identity of hot spots is a great tool for disrupting existing PPIs by introducing very few mutations. However, this is not sufficient for engineering new or altered PPIs for specificity or higher affinity, which has been a major goal of protein engineers from the beginning. For this, protein engineering methods have been developed along two main avenues: one is computational (i.e., use computer calculations to determine the needed composition), and the second is experimental, creating mutation libraries and selecting them for the desired trait. Next, we will shortly summarize progress in each of the two routes (see also Figure 1).

In 2018, the Nobel Prize in Chemistry was awarded for the development of the phage display method for the *in vitro* evolution of antibodies to bind any given target specifically. Phage display was first described in 1993 by Schreuder et al.^55^ It was the first of many other *in vitro* evolution methods that have since been developed. Over time, yeast display became the most widely used method for directed protein evolution, particularly for the development of high-affinity binders.^25^ Like other display methods, its principle is based on cycles of naïve protein library exposure, selection, and enrichment of yeast clones with desired properties. Yeast display, phage display, and ribosome display have proven to be effective methods for developing, improving, and altering activities of proteins for research, therapeutic, and biotechnology applications.^26^ Together with their relative ease of use and reasonable cost, the unprecedented power of these techniques have made them popular in many laboratories around the world. The most popular of these methods is yeast display due to its versatility, ease of use, low cost, and robust results for selecting binders for many proteins. Here, the use of *Saccharomyces cerevisiae* and its homologous recombination machinery reduces the need for laborious DNA library preparations, with only DNA fragments being needed.^27^ Coupling of the genotype–phenotype association with high-throughput single-cell analysis on a fluorescent activated cell sorter (FACS) offers a simple and efficient screening process.^25^ Due to their success, yeast display methodologies are constantly evolving.^28^ For example, a method was devised to select faster binding proteins through pre-equilibrium selection.^29^ Here, the prey and bait are only transiently incubated before selection, giving an advantage to faster (rather than tighter) binding proteins. Further method development was introduced by creating N- and C-terminal fusions to proteins with enhanced stability and fluorescence, accelerating the method and allowing even tighter binders to be selected.^15^

Library design has major implications on the outcome of the selection. To achieve complete coverage of all possible variants, the library size has to be 20 in the power of the number of amino acids of the protein (for example, 20^300^ for a small 100-amino acid, 300-nucleotide protein). This is obviously not feasible as the number surpasses the number of atoms in the universe (10^78^–10^82^). Yeast display libraries can include ≤10^9^ variants, while phage and ribosome display libraries can be composed of ≤10^12^ different variants. However, the actual number of correctly screened colonies is smaller due to transformation efficiency and analysis errors and thus further restricts the maximum variability of the library. This would allow for complete randomization of only a few residues. Therefore, much effort went into the design of smaller, more focused libraries. For example, methods have been developed to restrict either the positions or the amino acid mutations. For example, a library design with biased diversity in favor of Tyr/Ser/Gly residues but with the addition of small quantities of other amino acid types was sufficient to obtain many high-affinity antibodies against numerous antigens.^30^ Another example is restricting positions and mutations to only partial randomization.^31^ Still, these options limit the library toward variants of a very limited number of residues, which may not cover the full potential to obtain binding. For this, all amino acids of a protein should be mutated, as is done in natural evolution. This is usually done through error-prone polymerase chain reaction, which can be dialed to introduce two to four random mutations per protein. This would be sufficient to probe the complete protein with all possible mutations. However, this is not probing the effect of mutations that require two or three nucleotide changes to be reached (only 6–10 other amino acids are reached by single-nucleotide changes) or epistatic mutations, where each mutation on its own has a negative effect. To overcome this problem, one has to create multiple libraries on top of each other. A major problem here is that the intermediate species (single mutations) have reduced viability, due to either stability or lower affinity. Therefore, one has to probe the library through a path of least resistance and select many clones as the basis for the next library. Using this strategy, we recently succeeded in obtaining picomolar affinity binders of the receptor binding domain (RBD) of SARS-CoV-2 spike protein binding to ACE2.^15,32^ In summary, while selection methods have proven to be highly successful, they suffer from a limit of selecting epistatic mutations when there is high resistance of the intermediates^33^ (Figure 1A).

## Computational Tools for Engineering Protein–Protein Interactions

In 1987, Jeremy Knowles argued against the premature use of the word engineering, as we do not yet sufficiently understand proteins to engineer them.^34^ Since those early days, much has been learned, as recently reviewed.^35^ In the last critical assessment for protein structure prediction (CASP14, November 2020), an algorithm based on artificial intelligence, learning from known protein structures, successfully predicted to high resolution the structures of a majority of the test proteins, a transformative achievement suggesting that we now can predict the relation between sequence and structure.^36^ Surprisingly, it seems to be easier to design a *de novo* protein or compute a protein structure than it is to design binding sites from scratch or to predict binding sites. This was demonstrated by the results of the last critical assessment of predictions of interactions (CAPRI7, 2019),^37^ where docking predictions of the more difficult targets were problematic. Moreover, while computational docking is successful for the easier cases (where structural rearrangement is limited upon complexation), the prediction of a protein network purely by multiple docking computations of all against all is currently beyond the computational limit.^38^ Among others, this is due to the good docking solutions also found for nonbinders, which suggests that current force fields are not sufficiently good for providing exact solutions. In line with the better success in structure prediction than in binding predictions, designing proteins from scratch has by now become a doable task, with many examples given.^39^ One of the first successes in designing a new binder is the computational design of a protein targeting the conserved stem region of influenza hemagglutinin by the Baker group.^40^ The design principle was first to identify hot spot residues making energetically favorable interactions with the target surface and then to configure a scaffold based on an existing protein that anchors these energetically favorable interactions. In that case, the designed protein bound with very low affinity, which was enhanced by *in vitro* evolution using yeast display to nanomolar affinity. This approach was later enhanced, by using a large number of designed mini-proteins as a scaffold, reaching down to nanomolar affinity for influenza hemeagglutinin.^41^ An alternative approach for the rational design of *de novo* PPIs is the use of α-helices as the interfaces in *de novo* interactions. Here, one takes advantage of the well-known sequence–structure relationship of coiled coils, and indeed, this method has shown great promise^42^ (Figure 1B).

## Anti-COVID-19 Biologicals as a Test Case for PPI Engineering

COVID-19 was first reported in December 2019. As the virus generating this pandemic, SARS-CoV-2, is very similar (80% homologous) to the SARS-CoV virus that caused a pandemic in 2003, its primary mode of function was well understood. Within weeks, the complete sequence of SARS-CoV-2 was available, and structures of its main proteins appeared soon after. With the first step of infection being the binding of the spike protein of SARS-CoV-2 to ACE2 in human airways, the inhibition of this interaction became a prime target for drug development. This immediately generated a race among many of the leading groups with expertise in protein engineering to create such inhibitors. This provides us with an up-to-date and state-of-the-art reflection of the power of current technologies.

**Figure 2:**
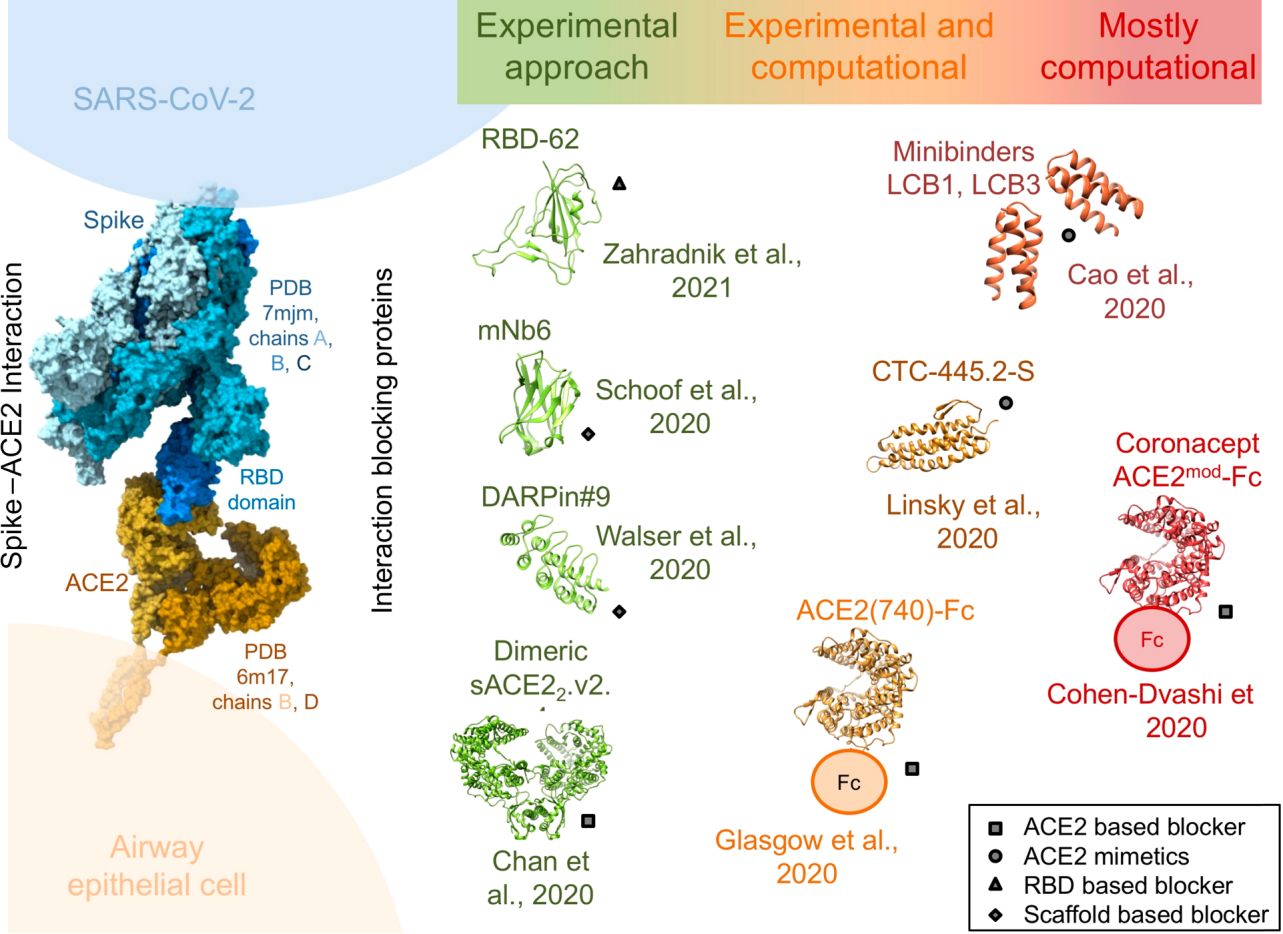
Anti-COVID-19 biologicals produced by protein engineering. The SARS-CoV-2 spike protein interacts predominantly with the ACE2 receptor on the surface of airways of epithelial cells. Blocking this interaction is a powerful way to inhibit viral replication. Different molecules using distinct strategies of actions were developed over the course of the first year of the SARS-CoV-2 pandemic. The molecules are organized with respect to the methodological approaches covering a continuum from exclusively experimental work (green) through mixed approaches (orange colors) to mostly computational work only (red).

Most groups targeted the spike protein binding motif (RBM) that interacts with ACE2, with few groups targeting ACE2 for blocking this interaction (Figure 2). With the ACE2 protein binding with a 10 nM affinity to the spike protein,^43^ the RBM of ACE2 was used by many groups either as a starting point to further enhance the affinity for spike or using the RBM on ACE2 as a template to generate new spike binding proteins. Also, the RBD of the spike protein was used as a template to enhance binding to ACE2, thus blocking the receptor from interacting with SARS-CoV-2. While the methods mentioned above used an existing naturally available template, other methods were template-free. Most prominent of these were the use of llama antibodies, called “nanobodies”, which are much smaller than human antibodies, more stable, and easier to use for binding selection, and DARPin molecules, which contain naturally occurring ankyrin repeat motifs that are used as a platform to rapidly evolve tight binders for therapeutic uses (Figure 2). We summarize several studies using each of the techniques below.

### Increasing the Affinity of ACE2 for the Spike Protein

Chan et al. aimed to generate an ACE2 decoy with a very high affinity for the RBD. For this, they created a library of all single mutations at the ACE2 binding site, transfected them to Expi293F cells, and selected for the RBD of SARS-CoV-2. From the enrichment ratio, they chose the best binding mutants, which were combined and re-selected for epistasis. This resulted in a 40-fold improvement over that of the wild type (WT), which after dimerization (providing avidity) resulted in a potent ACE2 decoy.^44^ While Chan et al. used *in vitro* evolution, Cohen-Dvashi et al.^45^ engineered a tight binding soluble ACE2 by computational design. To identify preferred residues for mutation, they made use of the deep mutational scan of the ACE2 interface binding the RBD^46^ and used 70 orthologous ACE2 genes with high sequence identity to human ACE2. The stability and binding energy toward SARS-CoV-2 of selected residues were calculated using Rosetta atomistic modeling.^47^ Residues identified as giving an advantage were combined. The enhanced ACE2 variant bound RBD with an affinity of 30 pM versus 9 nM for the WT. This resulted in an ∼30-fold improved inhibition (IC_50_) against viral entry. Glasgow et al. engineered a high-affinity ACE2 receptor using a combination of computational design, followed by *in vitro* evolution.^48^ First, they computationally designed the ACE2–RBD interface using Rosetta, including flexible protein backbone design, improving affinity by 12-fold. This was followed by *in vitro* evolution, using random mutagenesis and yeast surface display, achieving an overall 170-fold higher affinity compared to that of WT ACE2. After fusion to a human immunoglobulin crystallizable fragment to increase stability and avidity, they reached IC_50_s against viral entry of tens of nanograms per milliliter. Interestingly, while some mutations selected on ACE2 were similar among these three studies, many others were not, despite achieving a much higher affinity in all cases, confirming the plasticity of PPI interfaces. Instead of improving ACE2–RBD affinity, Guo et al. engineered WT ACE2 to form a trimer using a trimerization motif fused to ACE2.^49^ Trimerization increased avidity, resulting in picomolar binding to the spike protein, despite using WT ACE2. The trimer showed high neutralization efficacy toward SARS-CoV-2.

### Designing *De Novo* Nanoproteins Based on ACE2 for Spike Binding

The extracellular domain of ACE2 is a 650-amino acid protein, rich in N-glycosylations and S–S bonds, which requires expression in mammalian systems. Taking advantage of the known RBM on ACE2, two groups designed nanoproteins binding the spike RBD. Cao et al. used as a starting point computer-generated scaffolds resembling the ACE2 helix that interacts with the RBM.^50^ Creating a large number of designed peptides and sorting them by yeast display for binding resulted in binders with an ∼100 nM affinity. These were further optimized using *in vitro* evolution by yeast surface display, resulting in <100 pM affinity binders. These small inhibitors (∼60 amino acids) express well and inhibit SARS-CoV-2 entry with an IC_50_ of 0.15 ng/mL. Linsky et al.^51^ used the known binding site of ACE2 to design a small protein mimic. Still, as opposed to ref (50), the interface amino acids were not altered to avoid the escape of viral variants. Therefore, after the design, they used *in vitro* selection only of non-interface residues for binding optimization, resulting in a binding affinity similar to that of WT ACE2. Dimerization of the designed protein resulted in a low nanomolar affinity binder with good SARS-CoV-2 neutralization efficacy.

### Using Generic Scaffolds to Generate Spike Protein Binders

Schoof et al. took advantage of the by now well-established nanobody platform to devise an ultrapotent synthetic nanobody to neutralize SARS-CoV-2.^52^ Initial screening using yeast surface display of synthetic nanobodies resulted in micromolar affinity binders. These were trimerized and further matured by rounds of yeast display, resulting in a femtomolar affinity multivalent nanobody that locks the spike protein in an inactive conformation with picomolar neutralization activity. Ye et al. started with B cells isolated from a dozen non-immunized llamas and used them to construct a phage library, which underwent two rounds of selection against the RBD, increasing the affinity from 230 nM of the unselected library to 14 nM after selection.^53^ Fusion to FC further increased the binding affinity through avidity to 16 pM. The nanobody–FC complex showed good neutralization activity against SARS-CoV-2. Another generic scaffold is DARPin molecules, which contain naturally occurring ankyrin repeat motifs. The *in vitro* selections were done via ribosome display, which allows 10^12^ variants to be scanned. Walser et al.^54^ selected DARPins against three distinct epitopes of the S-protein, RBD, NTD, and S2, achieving nanomolar to picomolar affinity binders. The combination of those three resulted in potent SARS-CoV-2 neutralization.

### RBD Domain Protein Engineering

While most studies aimed to block the virus, Zahradnik et al. used the spike protein RBD to develop an inhibitor against ACE2.^32^ Using a newly devised yeast surface display method,^15^ and multiple rounds of selection (first to stabilize the RBD, then to increase the level of binding, and finally specifically to increase the RBD association rate), resulted in a picomolar binding inhibitor. The inhibitor had an IC_50_ of 10–200 pM against the different SARS-CoV-2 variants on VeroE6 cells and reduced the rate of SARS-CoV-2 infection in a hamster model.

## Summary

The success of generating many different SARS-CoV-2 inhibitors with picomolar IC_50_ within few months of the pandemic outbreak demonstrates that engineering of high-affinity binders is now a reality and that multiple methods are available to the designer (Figure 2). No study relied solely on computational methods. One study used previous deep sequence information;^45^ other studies used computation as the first step, followed by *in vitro* evolution, while some studies relied on only *in vitro* evolution. This shows that *in vitro* evolution methods are very powerful now, with new methods being devised all of the time, but this also indicates that computational methods came a long way and are now reliable resources for the engineering of protein binders. While for achieving the highest affinity one still needs *in vitro* evolution, the computational methods reduce the search space so that it can be managed by *in vitro* evolution methods.
